# Skeletal Mets From Squamous Cell Carcinoma of the Lower Limb: A Case Report

**DOI:** 10.7759/cureus.27155

**Published:** 2022-07-22

**Authors:** Yasmeen Alanazi, Asem Shadid, Salman T Almalki, Hatoon Bakhribah

**Affiliations:** 1 Department of Dermatology, King Fahad Medical City, Riyadh, SAU; 2 Department of Pathology, King Fahad Medical City, Riyadh, SAU; 3 Department of Oncology, King Fahad Medical City, Riyadh, SAU

**Keywords:** lower limb, immunotherapy, vitiligo, bone metastasis, squamous cell carcinoma (scc)

## Abstract

Squamous cell carcinoma (SCC) is the second most common non-melanoma skin cancer worldwide and one of the most capable of metastasis. The bone is a common metastatic site of cancers, which is a major cause of morbidity, with an estimated 350,000 people dying yearly from bone metastases. However, bone metastasis from an SCC in the lower limb is rarely reported in the literature and the role of systemic chemotherapy is not well established. We report a case of a 72-year-old gentleman with an SCC metastasis of the bone. This patient received six cycles of platinum-based chemotherapy with no surgical or radiation intervention.

## Introduction

Squamous cell carcinoma (SCC) is the second most common form of skin cancer in the world [1]. Most of the SCCs reported occur in sun-exposed areas, including the head, neck, chest, upper back, and hands [2]. However, SCC can occur in any location [3]. Lower extremity squamous cell carcinomas usually affect the anterior aspect of the lower limb and occur mostly in women and black individuals [3,4]. The incidence of nodule metastasis from SCC of the skin is approximately 4-5%, and 1.5% of individuals die from this disease [5,6]. The symptoms of SCC are highly variable, and the clinical presentation of invasive SCC is specific to the location [6,7]. Cutaneous SCC is among the most common cancers capable of metastasis [7]. However, bone metastases from an SCC in the lower limb are rarely reported in the literature.

## Case presentation

A 72-year-old male was known to have hypertension, ischemic heart disease post percutaneous coronary intervention (PCI), local prostate cancer, and deep vein thrombosis on subcutaneous heparin. The patient was on leuprolide and was stable with no recent elevation in prostate-specific antigen (PSA). The patient was also known to have vitiligo and did not seek any therapy for it. He presented with a right inguinal mass and ulcer for three months, and he did not have any history of trauma, burn, or previous surgery on this site. He was an ex-smoker. Upon examination, it was observed that he had a hard ulcerative mass of 5x4 cm in the right (RT) inguinal region along the crease with an irregular border, yellowish slough in the base, and blood oozing. The patient denied any development of vitiligo patches at the lesion site. Multiple hard enlargements of inguinal lymph nodes were found; sensation and distal pulsation were intact, and the systemic examination was normal.

**Figure 1:**
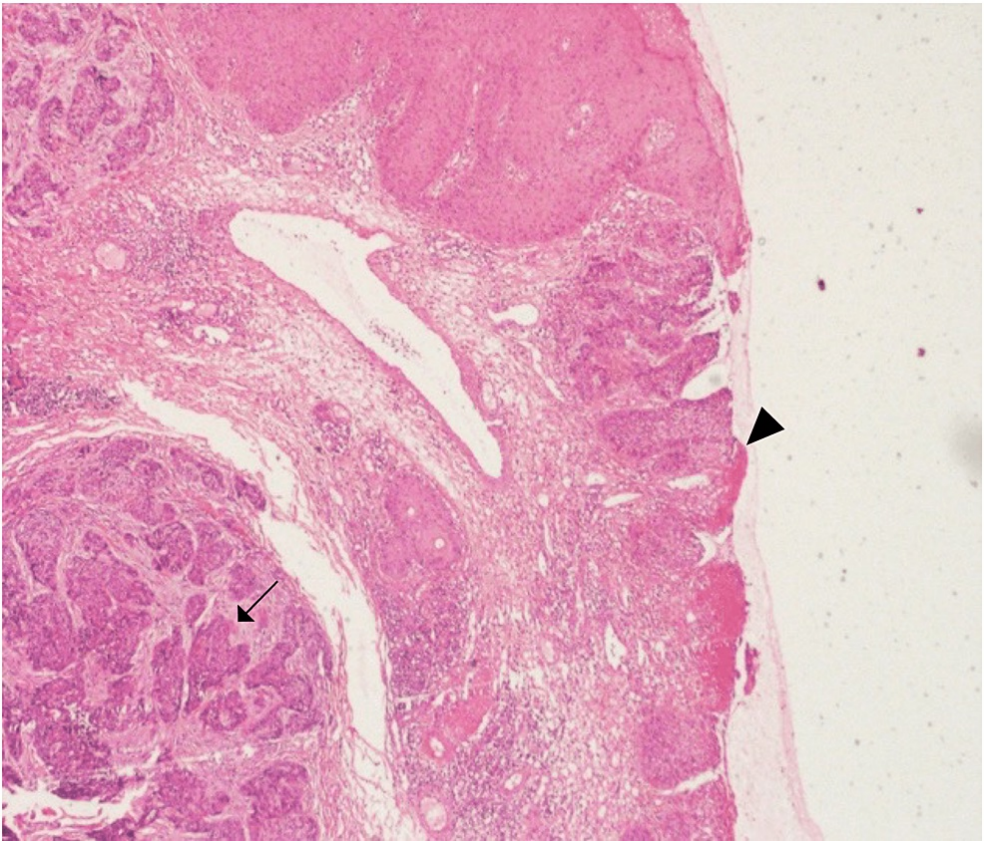
Squamous cell carcinoma nests invading the dermis (arrow). The overlying epidermis reveals squamous cell carcinoma in situ and ulceration (arrowhead) (H&E staining).

**Figure 2:**
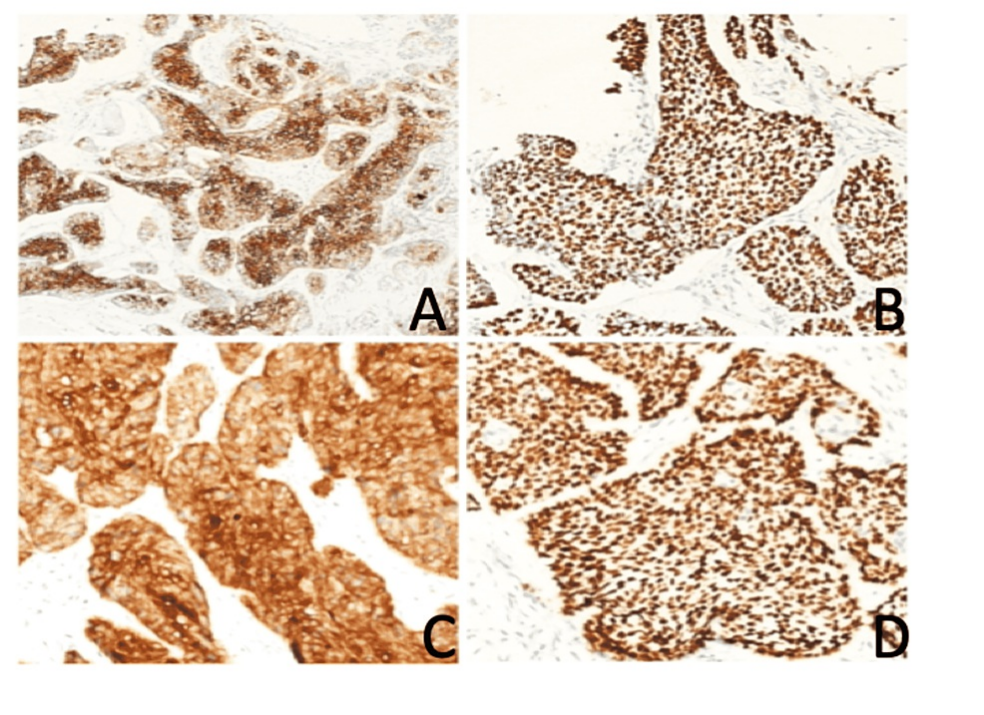
Immunohistochemical stain of the skin biopsy. The images show (A) squamous cell carcinoma positive for CK 5/6, (B) squamous cell carcinoma positive for P63, (C) squamous cell carcinoma positive for CK 5/6, and (D) squamous cell carcinoma positive for P63. CK: cytokeratin

An excisional biopsy was taken, which confirmed the presence of an infiltrating, non-keratinized well to a moderately differentiated squamous cell carcinoma (Figure 1). Immunohistochemical stain of the biopsy showed that the tumor cells were positive for cytokeratin (CK) 5/6 and P63, and were negative for CK 7, epithelial membrane antigen (EMA), carcinoembryonic antigen (CEA), human epidermal growth factor receptor 2 (HER-2), progesterone receptor (PR), estrogen receptor (ER), androgens (AR), gross cystic disease fluid protein 15 (GCDFP-15), S100, and Human Melanoma Black-45 (HMB-45) (Figures 2A-2D). Tumor marker levels for CEA, alpha-fetoprotein cancer antigen (CA) 19.9, and free PSA were normal.

**Figure 3:**
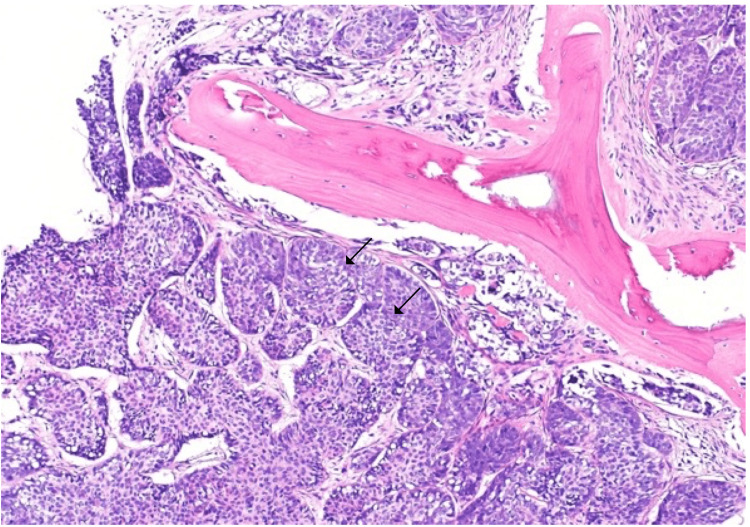
Metastatic squamous cell carcinoma of the bone (arrows) (H&E staining).

A positron emission tomography (PET)/CT scan revealed diffuse metastasis and an RT inguinal mass with standardized uptake value (SUV) max 9.6 (6.5x2.8 cm). Diffuse bone metastasis (mets) was observed in the left (LT) proximal femur SUV max 11.5, bilateral iliac, sacrum, vertebral bodies, RT scapula, RT upper ribs, and RT anterior pulmonary nodule 0.5x0.5 cm SUV max 4.7. No abnormal uptake was observed in the brain. US liver and biliary was normal. CT-guided spinal core biopsy was taken from the RT L5 pedicle to confirm that metastasis was from the SCC and not from prostate cancer (Figure 3).

The patient was diagnosed with stage 4 squamous cell carcinoma and received six cycles of platinum-based chemotherapy with no surgical or radiation intervention due to multiple sites of distant metastasis. The PET/CT scan was repeated after six months, which confirmed disease progression, after which the patient was started on immunotherapy nivolumab based on the case report and the series of refractory metastatic squamous cell carcinoma of skin with no surgical or radiation options.

A year after this treatment, the patient had multiple hospital admissions due to pneumonia. His condition deteriorated progressively and he was transferred into palliative care, where he was signed do-not-resuscitate (DNR) by three consultants. Unfortunately, the patient deceased one year and six months after being diagnosed.

## Discussion

Bone is one of the most common sites for metastatic cancer with an estimated mortality of 350,000 individuals annually. The most common sites of bone metastases are the spine, ribs, pelvis, proximal femur, and skull [8,9]. The exact incidence of bone metastasis is unknown. However, the incidences of distant metastases and mortality caused by cutaneous SCC have been reported [10].

Many risk factors that increase the probability of metastasis in SCC have been identified. These include immunosuppression, a tumor thickness >2.0 mm, a tumor size >2.0 cm, the tumor location, the level of differentiation, and a long duration of the primary lesion [7,10]. In this patient, the tumor size was 5 cm. Regarding treatments, the role of systemic chemotherapy for metastatic SCC is still unclear. However, previous studies have suggested that the most efficacious modalities are cisplatin, 5-FU, doxorubicin, bleomycin, or combinations thereof [10], as these have been shown to improve the progression-free survival percentage [10,11]. In our case, the patient received six cycles of platinum-based chemotherapy. There have been contradictory reports on whether there is an increased or equal risk of skin cancer in patients with vitiligo [12]. However, there has been a more recent systemic review supporting the claim that vitiligo may be protective against skin cancer [13].

## Conclusions

In conclusion, we reported a case of a 72-year-old male who initially presented with an ulcerated mass on his RT groin, which was diagnosed as cutaneous SCC. In this case, the most fatal complication was distant metastasis, as it progressed with chemotherapy and immunotherapy. This unusual presentation is extremely rare.
